# Support vector machine for classification of meiotic recombination hotspots and coldspots in *Saccharomyces cerevisiae *based on codon composition

**DOI:** 10.1186/1471-2105-7-223

**Published:** 2006-04-26

**Authors:** Tong Zhou, Jianhong Weng, Xiao Sun, Zuhong Lu

**Affiliations:** 1 State Key Laboratory of Bioelectronics, Southeast University, Nanjing 210096, China

## Abstract

**Background:**

Meiotic double-strand breaks occur at relatively high frequencies in some genomic regions (hotspots) and relatively low frequencies in others (coldspots). Hotspots and coldspots are receiving increasing attention in research into the mechanism of meiotic recombination. However, predicting hotspots and coldspots from DNA sequence information is still a challenging task.

**Results:**

We present a novel method for classification of hot and cold ORFs located in hotspots and coldspots respectively in *Saccharomyces cerevisiae*, using support vector machine (SVM), which relies on codon composition differences. This method has achieved a high classification accuracy of 85.0%. Since codon composition is a fusion of codon usage bias and amino acid composition signals, the ability of these two kinds of sequence attributes to discriminate hot ORFs from cold ORFs was also investigated separately. Our results indicate that neither codon usage bias nor amino acid composition taken separately performed as well as codon composition. Moreover, our SVM based method was applied to the full genome: We predicted the hot/cold ORFs from the yeast genome by using cutoffs of recombination rate. We found that the performance of our method for predicting cold ORFs is not as good as that for predicting hot ORFs. Besides, we also observed a considerable correlation between meiotic recombination rate and amino acid composition of certain residues, which probably reflects the structural and functional dissimilarity between the hot and cold groups.

**Conclusion:**

We have introduced a SVM-based novel method to discriminate hot ORFs from cold ones. Applying codon composition as sequence attributes, we have achieved a high classification accuracy, which suggests that codon composition has strong potential to be used as sequence attributes in the prediction of hot and cold ORFs.

## Background

Meiotic recombination occurs more frequently in some regions of the eukaryotic genome than in others, with variations of several orders of magnitude observed in frequencies of meiotic exchange per unit physical distance [1]. In *Saccharomyces cerevisiae*, meiotic recombination is initiated by double-strand DNA breaks (DSBs) [1,2]. Some genomic regions in which meiotic DSBs occur at relatively high frequencies are called hotspots, and by contraries, the regions with relatively low frequencies are called coldspots [2]. Although observations concerning individual hotspots and coldspots have given clues as to the mechanism of recombination initiation, the prediction of hotspots and coldspots from DNA sequence information is very limited [2]. So, several global mapping studies have been performed to map DSB sites on chromosomes in yeast to determine whether they share common DNA sequences and/or structural elements [2-5]. It was found that, in *S. cerevisiae*, the position of hotspots were nonrandomly associated with certain transcriptional profiles and some feature of chromosome structure related to GC-richness regions, while coldspots may associate with the centromeres and telomeres [2,6]. Further analysis showed that, in yeast, there is a significant correlation between codon usage bias and recombination rate, and the similar phenomenon was also observed in some other organisms, such as *Drosophila melanogaster*, mouse and human, which may be interpreted by biased genetic conversion during meiosis and/or Hill-Robertson interference [6-11].

Anyway, more mechanistic studies will be still critical in predicting hotspots and coldspots and defining corresponding operational rules [2]. Although experimental techniques can be applied for this purpose, they are laborious and time-consuming and therefore become infeasible for large numbers of genomic sequences [12]. Hence efficient and reliable computational methods for discriminating hotspots from coldspots are required.

An advanced method to this task employs statistical learning theory, typically the support vector machine (SVM), which is a type of supervised machine learning algorithm. Lin et al. have investigated the ability of SVM to discriminate ribosomal protein coding genes from all other genes of known function based on codon usage in *Escherichia coli*, *Mycobacterium tuberculosis *and *Saccharomyces cerevisiae *[13]. Codon usage was also formerly used by Friedel et al. as sequence attributes for separation of mixed plant-pathogen EST collections using SVM with high classification accuracy [12]. In this paper, we present a novel method for prediction of hot and cold ORFs located in hotspots and coldspots respectively in *S. cerevisiae *using SVM based on codon composition differences. Our method can accurately differentiate hot ORFs from cold ORFs, which suggests that codon composition is a satisfying sequence attribute. Moreover, our SVM based method was applied to the full genome: We predicted the hot/cold ORFs from all selected ORFs in yeast genome by using cutoffs of recombination rate and found that the performance of our method for predicting cold ORFs is not as good as that for predicting hot ORFs. Besides, in this study, we also detected a significant correlation between meiotic recombination rate and amino acid composition of certain residues in proteins encoded by the ORFs located in recombination hot or cold spots.

## Results and discussion

### Classification of hot and cold ORFs based on different sets of attributes

We use codon use frequency (FCU) to measure codon composition (see Methods section for details). As the input for the SVM, the FCU values of each ORF in *S. cerevisiae *were represented as a 61-dimensional vector. Ten-fold cross-validation was chosen to estimate performance of the SVM model. Table 1 indicates that, based on FCU, the SVM learning technique was able to accurately differentiate the hot ORFs from the cold ORFs with an accuracy of 85.0%, which suggests that the hot ORFs has a unique codon composition profile compared with the cold ORFs.

It was formerly suggested that the information contained in codon composition is representative of both codon usage bias and amino acid composition [13]. So, we evaluated these two attribute types separately on the same random training and test splits as above. In our study, codon usage bias and amino acid composition were measured by relative synonymous codon usage (RSCU) and amino acid use frequency (FAAU) respectively (see Methods section for details). Using only RSCU values as sequence attributes resulted in an average classification accuracy of 82.5%, while only considering amino acid composition for prediction, the SVM performed more poorly with an accuracy of 65.8% (see Table 1).

Compared with the original codon use frequency (FCU), neither RSCU nor FAAU contributed any new information for classification and these additional attribute sets taken separately did not have the same excellent performance as codon composition. And, according to McNemar's test, the FCU based method shows significant difference with respect to the other two methods (P < 0.05). Therefore, we can conclude here that codon composition has strong potential to be used as sequence attributes in the classification of hot and cold ORFs.

### Contribution of amino acid composition for classification

Since both amino acid composition and codon bias contribute to the uniqueness of the codon composition, now we were interested to determine the relative contribution of the amino acid composition signal to the ability of classification. So we took together the recombination rate and FAAU of each ORF into account. It was found that there were considerable positive correlations between recombination rate and the composition of positively charged amino acid His (r = 0.11, P < 0.05) and Arg (r = 0.13, P < 0.05), as well as significant negative correlations between recombination rate and the composition of polar amino acids Asn (r = -0.11, P < 0.05) and Ser (r = -0.12, P < 0.05), which means that there should be a marked enrichment in amino acids His and Arg whereas a notable depletion in Asn and Ser among the proteins coded by hot ORFs. To test the contribution of this skewed amino acid composition on the SVM classification, we trained our SVM models on all above sequence attributes separately again, excluding the amino acid His, Arg, Asn and Ser. The accuracies of these models, understandably, were not as good as those trained based on the data set without exclusion (see Table 2). However, it is evident that the accuracy of the model based on amino acid composition decreased much more significantly than those of the models based on FCU and RSCU. Moreover, applying McNemar's test, we detected a significant difference between the SVM model based on FAAU of all amino acids and the model with exclusion of the four amino acids mentioned above (P < 0.05), which strongly suggests the importance of this set of residues for the uniqueness of amino acid composition in both groups.

The difference in amino acid composition between the proteins coded by hot and cold ORFs probably reflects the structural and functional dissimilarity in these two groups. Gerton et al. have observed several correlations between hot or cold ORFs and gene functions: there was a very significant over-representation of the hotspot ORFs in the metabolism and ionic homeostasis functional classes, as well as an overrepresentation of coldspot ORFs in the categories of transport facilitation and intracellular transport, which may be due to an association of certain categories of genes with a particular chromatin structure that is favorable or unfavorable for initiating meiotic recombination [2]. At the same time, several previous studies have shown that proteins with similar function may share a similar amino acid composition [14-16]. Therefore, it is understandable that there is a skewed amino acid composition between the proteins coded by hot and cold ORFs. However, why amino acid composition is associated with the variation in meiotic recombination rate has not been solved unambiguously.

### Contribution of codon bias for classification

**Figure 1:**
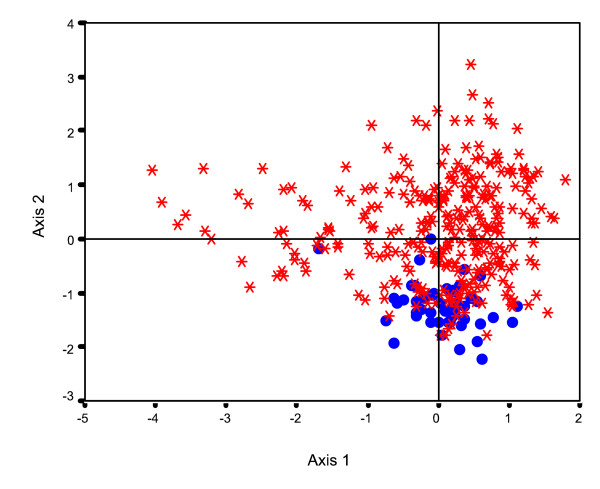
**Plot of the two most dominant axes generated by PCA**. Hot ORFs are represented by the red asterisks, while cold ORFs are represented by the filled blue circles.

To determine the relative contribution of the codon bias signal to the ability of SVM to distinguish the hot ORFs from the cold ORFs, we conducted a principal component analysis (PCA) of RSCU values on all selected ORFs. Figure 1 shows the position of the ORFs on the plane defined by the first and second major axes generated by PCA. The two major axes account for 15.9% and 12.5% of all variation of codon usage bias among genes respectively, whereas the other axes account for no more than 4%. It is evident that the second axis discriminates the genes in both hot and cold groups, although there is a considerable overlap between the two clusters: almost all cold ORFs cluster in the lower quadrants while majority of the hot ORFs are located in the upper quadrants.

In *S. cerevisiae*, it was thought that the codon usage bias strongly correlates with gene expression [17]. In this study, we used the codon adaptation index (CAI) to measure the gene expression level [18]. High CAI genes are presumed to be highly expressed while low CAI genes are presumed to be lowly expressed. As observed in previous studies, we found that there is a significant negative correlation between the first axis and CAI (r = -0.92, P < 0.00001), which means that the first axis can discriminate genes with different expression level [17]. This kind of correlation between codon usage and gene expression reflects the nature selection acting at translational level.

Besides, a considerable positive correlation was also found between the second axis and recombination rate (r = 0.39, P < 0.00001) in yeast. This kind of correlation was also observed in *D. melanogaster*, which has been explained by two proposed models [7-9]. In the first model, it is proposed that the reduction of codon bias in the regions with limited recombination is consistent with Hill-Robertson interference [7,19]. The effectiveness of nature selection on one target due to stochastic effects of selection on linked targets is expected to be reduced by this conflict [6]. Computer simulation also suggests that the effect of genetic linkage should be particularly damaging in the case of weak selection, including the selection acting on codon usage [8,9,19,20]. The second model proposes that the correlation between codon usage pattern and recombination rate is caused by recombination-related mutational bias [8,9]. There is a positive correlation between G+C content and recombination rate in the organisms, such as *D. melanogaster*, *S. cerevisiae*, mouse and human [2,6-11], which probably can be interpreted by biased genetic conversion during meiosis [9].

**Figure 2:**
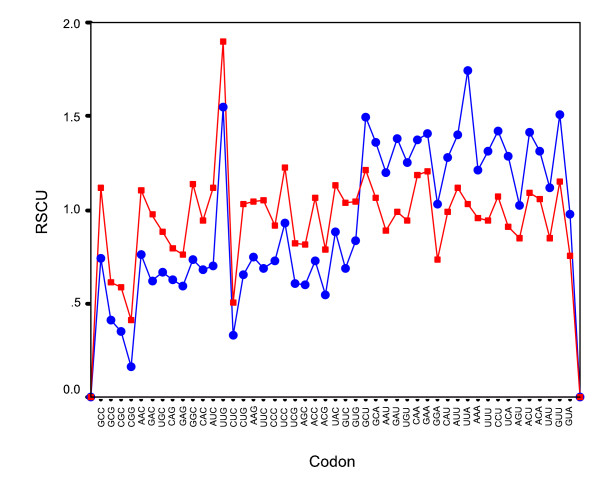
**A comparision of the average RSCU values of hot ORFs with that of cold ORFs**. From left to right, the first 26 codons are statistically more frequent in the hot ORFs while the last 21 triplets are used at the higher frequency in the cold ORFs (P < 0.05). The RSCU values of hot group are marked with filled red squares, while those of cold group are marked with filled blue circles.

To examine whether Hill-Robertson interference has significant influence on the codon usage in yeast, the RSCU values in the hot and cold data sets was compared using χ^2 ^test. Several previous studies have shown that nearly half of the translationally preferred codons are ended in G or C in this organism [6,21,22]. So, if Hill-Robertson really plays an important role in shaping the codon usage, the preferred codons in the hot data set should contain the codons not only end in G or C, but also end in A or U, because the selection on hot spots is thought to be more effective than on cold spots due to Hill-Robertson interference. In present study, 26 codons, for 18 amino acids, were identified as significantly (P < 0.05) more frequent in the hotspot ORFs while another 21 triplets were used at the higher frequency in the coldspot ORFs (shown in Figure 2). However, in the 26 codons which are preferred in the hot ORFs, none is A or U ended translationally preferred codon, which suggests that Hill-Robertson interference only partially accounts for the correlation between codon bias and recombination rate in *S. cerevisiae*.

In fact, there is a significant positive correlation between the frequency of G+C at the synonymous third codon position (GC3_S_) and the second axis (r = 0.96, P < 0.00001). At the same time, the recombination rate for each ORF is greatly positively correlated with GC3_S _in yeast (r = 0.40, P < 0.00001). Given that GC3_S _may reflect regional base compositional bias, the second model we mentioned above might explain the association between codon usage and recombination rate much better. In other words, biased genetic conversion between parental chromosomes during meiosis might mainly account for the correlation between codon usage variation and recombination in *S. cerevisiae*.

In addition, we have mentioned above that there are correlations between hot or cold ORFs and gene function. And it has also been reported that there is a relationship between gene function and codon usage pattern in eukaryotic organisms [23,24], which may partially account for the correlation between codon bias and recombination rate in yeast.

To evaluate the contribution of the codons listed in Figure 2 on the SVM classification, we trained our SVM models only on codon composition and codon usage bias of these 47 codons respectively again. As expected, there was no significant drop in accuracy whether we applied FCU or RSCU values as sequence attributes (see Table 3). Moreover, according to McNemar's test, we didn't detect any significant difference between the original SVM models trained on FCU or RSCU values without exclusion and the corresponding models based only on the 47 codons respectively, which proves the importance of these key codons for classification.

### Applying the SVM based method to full genome analysis

Our above analysis is only focused on the ORFs located in hot and cold spots which were detected by Gerton et al. Now, we will take all the ORFs in *S. cerevisiae *genome into account, including the neutral ORFs.

**Figure 3:**
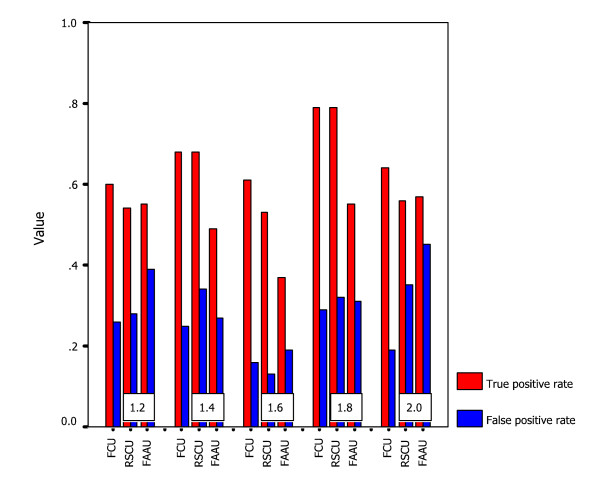
**The performance of SVM for predicting hot ORFs from the full genome**. The numerical value stuck on each bar cluster denotes the recombination rate cutoff of hot ORFs.

To examine the ability of our SVM based method for identifying hot ORFs from the full genome, we set a cutoff to measure recombination rate. The ORFs with relative recombination rate greater than the given cutoff were regarded as hot ORFs and positively labelled, while the other ORFs were negatively labelled. All the parameters of SVM were the same as our above analysis. Ten-fold cross-validation was used for performance estimation. Figure 3 shows the true positive rate and false positive rate of our method applying to the full genome at 5 different cutoffs. It is evident that when using FCU as sequence attribute, the performance of SVM model is better than the models based on RSCU and FAAU at each cutoff. Especially at the cutoff 1.8, the true positive rate is near 80% while the false positive rate is less than 30%.

**Figure 4:**
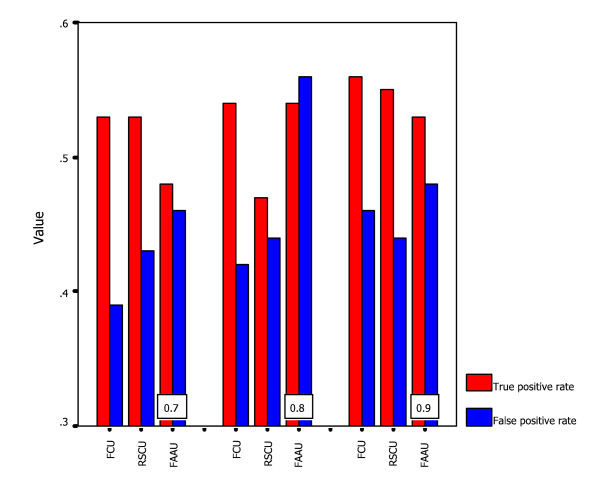
**The performance of SVM for predicting cold ORFs from the full genome**. The numerical value stuck on each bar cluster denotes the recombination rate cutoff of cold ORFs.

We also examined the ability of our method for detecting cold ORFs from the full genome. We classified the ORFs as either cold or non-cold by using a cutoff on the measured recombination rate. The ORFs with relative recombination rate less than the given cutoff were regarded as cold ORFs and positively labelled, while the other ORFs were negatively labelled. Ten-fold cross-validation was used for performance estimation again. Figure 4 shows the true positive rate and false positive rate of our method for detecting the cold ORFs from the full genome at 3 different cutoffs. Although the FCU based model behaves better than the models based on RSCU and FAAU, it is obvious that the performance of our SVM based method for predicting cold ORFs is not as good as that for predicting hot ORFs, which may imply that it is more difficult to detect coldspots than to detect hotspots using computational method. The detailed predicted results when using cutoffs 1.2 and 0.8 to measure hot and cold regions respectively are listed in Additional file 1.

## Conclusion

Prediction of meiotic recombination hot/cold spots in eukaryotic genomes based on computational technique is a challenging problem, partially because of current limited scale of published experimental data and few models to represent the training data. In this paper, we have introduced a SVM-based novel method to discriminate hot ORFs from cold ones. Using codon composition as sequence attributes, we have achieved a high classification accuracy. Since codon composition is a fusion of codon usage bias and amino acid composition signals, the ability of these two kinds of sequence attributes for classification was also investigated separately. Our results indicate that neither codon usage bias nor amino acid composition taken separately performed as well as codon composition.

Moreover, our SVM based method was also applied to the full genome: We tried to predict the hot/cold ORFs from all selected ORFs in yeast genome by using cutoffs of recombination rate. We found that the FCU based model still behaved better than the models based on RSCU and FAAU. However, the performance of our method for predicting cold ORFs is not as good as that for predicting hot ORFs.

In addition, we also observed a considerable correlation between meiotic recombination rate and amino acid composition of positively charged residue His and Arg and polar residue Asn and Ser in proteins encoded by the ORFs located in hot/cold spots, which probably reflects the structural and functional dissimilarity between the hot and cold groups.

## Methods

### Sequence data

Gerton et al. have estimated relative recombination rates for most of the *S. cerevisiae *loci using DNA microarrays at a resolution of about 2–3 kb [2]. In *S. cerevisiae*, the average length of intergenic regions is about 500 bp. Therefore, although most hotspots are intergenic rather than intragenic, ORFs are used to locate hot/cold spots by Gerton et al. They detected 303 hot ORFs clustered into 177 hotspots whose recombination rates ranked in the top 12.5% and 49 cold ORFs clustered into 40 coldspots whose recombination rate ranked in the bottom 12.5% [2]. We extracted the hot/cold ORFs (one of the cold ORFs listed in Gerton's paper doesn't exist) and other neutral ORFs from GenBank database. The corresponding recombination data were obtained from [25] which was generated by Gerton et al. The relative recombination rate of each ORF is determined by the median array value of seven microarray experiments.

### Sequence attributes

Codon composition was measured by codon use frequency (FCU). Each ORF was represented by 61-dimensional vector with respect to the 61 sense codons. The FCU value of the j^th ^codon for the i^th ^amino acid was calculated thus:

*FCU*_*ij *_= *obs*_*ij*_/*Total*

where obs_ij _is the observed number of the j^th ^codon for the i^th^amino acid and Total is the total number of codons in the ORF. FCU is inherently the fusion of both codon usage bias and amino acid composition signals [13].

To examine synonymous codon usage without the confounding influence of amino acid composition of different gene samples, the values of relative synonymous codon usage (RSCU) of different codons in each sequence were also calculated. The RSCU value of the j^th ^codon for the i^th ^amino acid was calculated by

RSCUij=obsij(∑k=1niobsik)−1ni
 MathType@MTEF@5@5@+=feaafiart1ev1aaatCvAUfKttLearuWrP9MDH5MBPbIqV92AaeXatLxBI9gBaebbnrfifHhDYfgasaacH8akY=wiFfYdH8Gipec8Eeeu0xXdbba9frFj0=OqFfea0dXdd9vqai=hGuQ8kuc9pgc9s8qqaq=dirpe0xb9q8qiLsFr0=vr0=vr0dc8meaabaqaciaacaGaaeqabaqabeGadaaakeaacqWGsbGucqWGtbWucqWGdbWqcqWGvbqvdaWgaaWcbaGaemyAaKMaemOAaOgabeaakiabg2da9iabd+gaVjabdkgaIjabdohaZnaaBaaaleaacqWGPbqAcqWGQbGAaeqaaOWaaeWaaeaadaaeWbqaaiabd+gaVjabdkgaIjabdohaZnaaBaaaleaacqWGPbqAcqWGRbWAaeqaaaqaaiabdUgaRjabg2da9iabigdaXaqaaiabd6gaUnaaBaaameaacqWGPbqAaeqaaaqdcqGHris5aaGccaGLOaGaayzkaaWaaWbaaSqabeaacqGHsislcqaIXaqmaaGccqWGUbGBdaWgaaWcbaGaemyAaKgabeaaaaa@5264@

where obs_ij _is the observed number of the j^th ^codon for the i^th^amino acid which has n_i _type of synonymous codons. This attribute is independent of amino acid usage and it is obvious that RSCU value close to 1.0 indicates a lack of bias for the corresponding codon [26].

As well as the definition of FCU, amino acid use frequency (FAAU) was used to examine the amino acid composition of the corresponding protein for the selected ORF. The FAAU value of the i^th ^amino acid was calculated as below:

*FAAU*_*i *_= *obs*_*i*_/*Total*

where obs_i _is the observed number of amino aicd i in protein sample, and Total is the total number of the twenty kinds of amino acids in the protein.

### Support vector machine

Support vector machine (SVM) is a machine learning technique based on statistical theory. The principle of SVM is to find a maximum margin hyperplane for classification. If it is not possible in the given space, the instances are mapped to a higher dimensional space using the kernel function. Kernel function allows one to work in a higher dimensional space without computing all elements, which reduces computational complexity and connects the input space and the higher dimensional space directly. SVM will then choose a maximum soft margin separating hyperplane in this higher dimensional space, which separates the training instances by their classes. The classification of a test sample will then be determined by a sign function which is defined by the parameters of the hyperplane. The instances closest to the hyperplane are called support vectors and are vital for training [27]. Using SVM requires careful attention to the kernel function and the magnitude of the trade-off between accuracy and generalization [13].

In this paper, we used SVM^light ^version 6.01 for data training and classifying. To ensure that the parameter estimation and model generation of SVM is completely independent of the test data, the original data set of hot and cold ORFs was divided into two parts: set A and B. Set A was used as a separate validation set to optimize the parameters of SVM, which contains one third of the original sequences (117 ORFs). The remainder sequences were put into set B for performance evaluation (234 ORFs). When classifying hot and cold ORFs, the SVM models were trained with all the hot ORFs with positive labels and all the sequences from coldspots with negative labels. Ten-fold cross-validation was used for both parameter and accuracy estimation. Ten-fold cross-validation is to divide the dataset of hot and cold ORFs randomly into ten subsets and then alternately using one subset for testing and the other nine sets for training. In our study, ORFs from the same hot or cold spot were placed in one subset. In other words, we didn't distribute the ORFs from the same spot to different subsets.

We used different kernel functions in our experiments, including linear function, polynomial function and radial basis function. To obtain SVM classifier with optimal performance, the penalty parameter C and the parameters of kernel function are tuned by the standard grid search method based on set A. In this study, the value of parameter C was optimized to 100. When employing FCU as sequence attributes, the best results were obtained using the radial basis function kernel with γ = 200; while employing RSCU and FAAU as sequence attributes, the best performance was obtained using the polynomial kernel of five and three degree respectively, which implies that the FCU based classification problem is much more non-linear than the problems based on RSCU and FAAU.

### Measurement of SVM performance

The performance of the SVM model in distinguishing hot ORFs from cold ORFs was evaluated by ten-fold cross-validation using sensitivity, specificity, overall performance (OP) and accuracy. These indices are determined thus:

Sensitivity = TP/(TP + FN)

Specificity = TN/(TN + FP)

OP = (Sensitivity + Specificity)/2

Accuracy = (TP + TN)/(TP + TN + FP + FN)

where TP, TN, FP and FN are the number of true positives, true negatives, false positives and false negatives respectively.

When we examined the ability of our SVM based method to identifying hot or cold ORFs from the full genome, true positive rate and false positive rate are used for performance evaluation.

True positive rate = TP/(TP + FN)

False positive rate = FP/(TN + FP)

McNemar's test was also applied to decide whether significant differences exist between the performances of any two SVM models based on different attribute types. This test is performed by summarizing the classification errors of the two algorithms and has a lower Type I error (the probability of incorrectly detecting a difference when no difference exists) [28].

### Principal component analysis

To assess the statistical difference between the hot and cold ORFs, principal component analysis (PCA) was used to investigate the major trends in codon usage variation among genes. The RSCU values of each ORF were plotted in a multidimensional space of 59 axes (excluding Met, Trp and stop codons) and PCA identified a series of new orthogonal axes accounting for the greatest variation among genes. The analysis yielded the coordinate of each ORF on each new axis, and the fraction of the total variation was accounted for by each axis.

## Authors' contributions

TZ participated in the design of the study, performed the statistical analysis and drafted the manuscript. JW participated in the design of the study and set up computational facility. XS extensively edited the manuscript and made many important changes. ZL conceptualized the project, conceived of the study, participated in its design and helped to draft the manuscript. All authors read and approved the final manuscript.

## Supplementary Material

Additional File 1 A list of all predicted cold/hot regions calculated for whole genome by ten-fold cross-validationThe cutoffs of recombination rate for hot and cold regions are 1.2 and 0.8 respectively. Exp is experimental recombination rate (we defined the recombination rate for a given locus as the median of all available measures for that locus). P_hot and p_cold are predicted values. Click here for file

## Figures and Tables

**Table 1:** The performance of SVM for differentiating the hot ORFs from the cold ORFs. The SVM models were trained using codon composition, codon bias and amino acid composition as sequence attributes respectively.

Attributes	Sensitivity (%)	Specificity (%)	OP (%)	Accuracy (%)
FCU	86.6	75.0	80.8	85.0
RSCU	84.2	71.9	78.1	82.5
FAAU	66.3	62.5	64.4	65.8

**Table 2:** The performance of SVM with exclusion of certain amino acids. The SVM models were trained using codon composition, codon bias and amino acid composition as sequence attributes respectively, excluding His, Arg, Asn and Ser from the training datasets.

Attributes	Sensitivity (%)	Specificity (%)	OP (%)	Accuracy (%)
FCU	86.1	68.8	77.5	83.8
RSCU	84.2	65.6	74.9	81.6
FAAU	61.4	50.0	55.7	59.8

**Table 3:** The performance of SVM only trained on the key codons. The SVM models were trained using FCU and RSCU values of the codons listed in Figure 2 as sequence attributes respectively. These codons are thought to play important roles in classification.

Attributes	Sensitivity (%)	Specificity (%)	OP (%)	Accuracy (%)
FCU	86.6	68.8	77.7	84.2
RSCU	84.6	65.6	75.4	82.1
